# Collaborative Primary-Care Workforce Models: An Integrative Review of Evidence Informing RN Prescriber Integration with Family Physicians and Nurse Practitioners

**DOI:** 10.3390/healthcare14131899

**Published:** 2026-06-30

**Authors:** Tomasz Karczewski, Dawid Karczewski, Merjorie M. A. Pinero, Avni K. Patel

**Affiliations:** Cranston Ridge Medical Clinic, Calgary, AB T3M 3A9, Canada; tomasz@cranstonridgemedical.com (T.K.); merjorie@cranstonridgemedical.com (M.M.A.P.); avni@cranstonridgemedical.com (A.K.P.)

**Keywords:** registered nurse prescribing, non-medical prescribing, integrative review, primary care, family physician, nurse practitioner, interprofessional collaboration, team-based care, medication management, access to care, clinical governance

## Abstract

**Background/Objectives**: Registered nurse (RN) prescribing is increasingly discussed as a strategy to improve primary-care access, medication follow-up, chronic disease management, and service responsiveness. The available evidence, however, does not directly test a single coordinated RN prescriber–family physician/nurse practitioner (FP/NP) model. This integrative review synthesized heterogeneous evidence relevant to how RN prescribing may be organized within team-based primary care. **Methods**: A structured integrative review approach was used to map evidence from nurse and non-medical prescribing, RN-led primary care, nurse–physician substitution, interprofessional collaboration, chronic disease medication titration, patient-experience, and implementation research. Searches completed on 30 March 2026 included PubMed/MEDLINE, PubMed Central, the Cochrane Library search interface, publisher full-text platforms, targeted scholarly searches, citation chasing, and Canadian regulatory/professional sources. Methodological quality was appraised using AMSTAR 2- and CASP-informed criteria, and the strength of interpretation was assessed narratively. No meta-analysis was performed because of substantial heterogeneity and the risk of double-counting primary studies included in prior evidence syntheses. **Results**: A total of 286 records were identified. After de-duplication, screening, and eligibility assessment, 37 peer-reviewed records were included: 30 review-level or evidence-synthesis records and 7 primary, mixed-methods, or patient-experience studies. Four official regulatory/professional sources were retained separately for context. Nurse and non-medical prescribing were generally associated with comparable or favourable outcomes for blood pressure, glycated hemoglobin, low-density lipoprotein cholesterol, medication adherence, patient satisfaction, and selected access outcomes in defined contexts. Direct evidence for the exact RN prescriber–FP/NP configuration remains limited. **Conclusions**: Current evidence is consistent with a coordinated RN prescribing model embedded within primary-care teams, but does not establish causal superiority of this configuration over other models. Coordinated RN prescribing should therefore be understood as an evidence-informed and testable implementation model requiring prospective evaluation, particularly for diagnostic safety, adverse events, continuity, workload, cost, and patient-level outcomes.

## 1. Introduction

Primary care is expected to deliver timely acute assessment, prevention, chronic disease management, medication renewal, diagnostic testing, and longitudinal coordination at a time of workforce shortages and increasing patient complexity. In this context, RN prescribing has been advanced as one potential mechanism for improving access and redistributing work across the primary-care team. The practical question is not only whether RNs can prescribe, but how RN prescribing should be designed so that gains in access are not made at the expense of diagnostic safety, medication continuity, or accountability for follow-up.

A primary-care patient rarely experiences prescribing as an isolated professional act. Prescribing is embedded in triage, diagnosis, medication reconciliation, monitoring, safety-netting, laboratory interpretation, management of multimorbidity, and escalation when a presentation is outside the scope of a defined pathway. For that reason, RN prescribing may be most relevant to primary-care service design when considered together with the roles of family physicians and nurse practitioners (NPs), who often function as comprehensive longitudinal primary-care prescribers and escalation partners. Treating family physicians and NPs as coordination partners for the purposes of this review does not imply identical training, regulation, scope of practice, or clinical authority; rather, it reflects their shared operational role in many primary-care settings as clinicians who can support diagnostic uncertainty, complexity, continuity, and higher-risk prescribing.

Internationally, nurse prescribing remains associated with recurring debates about role boundaries, diagnostic responsibility, training requirements, legal liability, prescribing autonomy, medical oversight, patient understanding, interprofessional trust, and whether expanded nursing roles improve access without simply redistributing workload or risk. These debates are particularly important in primary care, where a medication decision may be inseparable from diagnosis, follow-up responsibility, abnormal-result management, and continuity [1,2,3,4,5].

Alberta is used in this review as a contemporary policy example rather than as the sole intended scope of the article. Documents from the College of Registered Nurses of Alberta (CRNA) indicate that RNs with prescribing authorization can prescribe Schedule 1 drugs, except for controlled drugs and substances, and can order diagnostic tests in an identified area of clinical practice when necessary standards, competencies, and clinical support tools are met [6,7,8]. The Canadian Nurses Association framework similarly locates RN prescribing as an access and system-design innovation that requires attention to education, regulation, liability, interprofessional relationships, organizational readiness, and public awareness [9].

The relevant evidence is spread across the literature on nurse prescribing and non-medical prescribing, RN-led primary care, nurse–physician substitution, nurse practitioner and family physician collaboration, chronic disease medication titration, patient satisfaction and concordance, medication safety, and implementation research. A focused review on Canadian RN prescribing alone would miss much of the transferable evidence; a broad review of all non-medical prescribing might lose sight of the specific primary-care coordination question. An integrative review design was therefore chosen to synthesize evidence across related domains while remaining explicit about indirectness and limits of inference.

The purpose of this review was to synthesize evidence related to RN prescribing as practiced within team-based primary care and to assess the extent to which such prescribing might be expected to affect access, clinical outcomes, patient experience, safety, continuity, resource use and implementation quality when used in close coordination with a family physician or NP. This review does not presuppose that a coordinated RN prescriber–FP/NP model has been established as a preferred approach to primary-care workforce design. Rather, it assesses whether the existing evidence base supports testing this model as a feasible and potentially promising approach.

## 2. Materials and Methods

### 2.1. Review Design and Reporting Approach

An integrative review design was used because the review question required synthesis of different types of evidence, including systematic reviews, randomized and quasi-experimental studies, observational studies, qualitative syntheses, implementation studies, and regulatory/professional documents. Integrative reviews are used when a clinically important question cannot be answered by a single homogeneous body of intervention trials alone and when conceptual, implementation and service-delivery evidence must be considered together [10].

This review involved a structured search, explicit eligibility framework, data-charting form, critical appraisal process, and transparent narrative synthesis. A working review plan was developed before the final synthesis and specified the review question, eligibility framework, search concepts, information sources, screening process, data items, appraisal approach, and synthesis plan. The working plan was internal and was not registered in PROSPERO, the Open Science Framework, or another public repository; it was not publicly posted, which precludes external verification of a priori decisions and is a recognized methodological limitation.

The review was not intended to produce a pooled effect estimate or formal certainty rating for a single intervention–comparator–outcome structure because it was an integrative review, not a protocol-driven systematic review or meta-analysis. Accordingly, systematic-review elements such as registry registration, full systematic-review reporting compliance, formal GRADE assessment, statistical heterogeneity testing, and sensitivity analyses were not applied as governing requirements. Where useful, transparent elements such as source-selection counts, a flow diagram, explicit eligibility criteria, and structured appraisal were retained.

The guiding question was as follows: what evidence is available to inform RN prescribing in primary-care settings when RN prescribers are coordinated with family physicians or NPs, and what does this evidence suggest about access, patient outcomes, patient experience, safety, continuity, resource use, and implementation conditions?

### 2.2. Eligibility Framework

The eligibility framework used to guide source inclusion and exclusion is summarized in Table 1. The criteria were deliberately broad because the review was designed as an integrative synthesis of evidence relevant to RN prescribing, primary-care workforce organization, implementation, and team-based care.

### 2.3. Information Sources and Search Approach

The final structured search date for all reported electronic, targeted scholarly, citation, regulatory, and professional source searches was 30 March 2026. Data sources included PubMed/MEDLINE-indexed records, PubMed Central full-text records, the Cochrane Library search interface, publisher full-text platforms for relevant journals, targeted scholarly searches, reference lists of included reviews and primary studies, and official Canadian and Alberta regulatory or professional sources. Official sources were retained for contextual interpretation and were not treated as intervention-effect evidence unless they reported original data.

The search was designed to identify high-yield evidence across related domains rather than to exhaustively retrieve every record across all health-science subscription databases. This design choice is consistent with the integrative nature of the review but limits comprehensiveness compared with a protocol-driven systematic review. Searches in Embase, CINAHL, Scopus, and Web of Science were not independently reported as stand-alone database searches. A complementary CINAHL search was considered because of the database’s relevance to nursing literature, but an independently auditable CINAHL search could not be completed for this revision. This limitation is particularly relevant because CINAHL may index nursing-specific qualitative studies, role-development papers, implementation studies, education-focused literature, and professional-practice research that are less visible in PubMed/MEDLINE or publisher searches. Rather than reporting an unverified search, the potential consequences of not searching CINAHL and other databases are addressed explicitly in the limitations.

Search concepts combined terms for nurse prescribing and non-medical prescribing, primary care, team coordination, family physicians or general practitioners, NPs, patient outcomes, safety, access, chronic disease medication titration, and implementation. The full search strategy, source limits, and search-date details are provided in Supplementary Files S1–S4.

### 2.4. Source Selection and Data Charting

Records were screened in two stages: title/abstract or search-snippet screening, followed by full-text or full-record assessment when the source appeared potentially eligible. Two reviewers screened candidate records. Disagreements or uncertainties were resolved through discussion and, when necessary, by consulting the wider author team. Cohen’s kappa was not calculated because the review used an iterative integrative approach rather than a fully blinded duplicate systematic-review screening process; this is acknowledged as a limitation.

Data were charted using a structured form that captured study design, setting, population, nurse role, prescribing or medication-management model, comparator or context, coordination with family physicians, general practitioners or NPs, patient outcomes, service outcomes, patient experience, safety, implementation factors, limitations, and interpretation for primary-care team design. Initial data charting was performed using the structured form and was then checked for consistency against the eligibility framework and synthesis domains. No automated eligibility decisions were used.

### 2.5. Critical Appraisal and Strength of Interpretation

Methodological quality and risk-of-bias considerations were appraised using design-appropriate criteria informed by AMSTAR 2 for systematic reviews and CASP tools for qualitative, mixed-methods, observational, and primary studies [11,12]. Because the evidence base included heterogeneous study designs and many review-level sources, formal certainty ratings were not applied. Instead, the strength of interpretation was summarized narratively using the following considerations: evidence type, study design, transparency of methods, risk-of-bias or methodological limitations, consistency across sources, directness to coordinated RN prescriber–FP/NP primary-care models, precision or outcome specificity, and completeness of safety, cost, workload, and implementation reporting.

The appraisal was used to interpret the relative contribution of each source to the synthesis, to separate stronger or more direct evidence from indirect or contextual evidence, and to identify where conclusions were limited by mixed prescriber groups, overlapping reviews, incomplete safety reporting, or context-specific implementation evidence. It was not used to generate a pooled effect estimate, a formal GRADE certainty rating, or a ranking of the proposed model as empirically superior. Study-level methodological appraisal and an evidence-hierarchy summary are provided in Supplementary Files S1–S4.

### 2.6. Synthesis Methods and Rationale Against Meta-Analysis

Integrative narrative synthesis was selected because the evidence differed in jurisdiction, prescriber role, training requirements, autonomy, clinical setting, comparator, outcome definition, follow-up period, and healthcare-system context. Many included sources were systematic reviews or scoping reviews that themselves contained overlapping primary studies; pooling their reported estimates would have risked double-counting and would not have produced a clinically interpretable estimate for the specific coordinated RN prescriber–FP/NP model. For these reasons, no meta-analysis, funnel plot, small-study-effect assessment, formal statistical heterogeneity analysis, statistical subgroup analysis, or statistical sensitivity analysis was performed.

The synthesis was organized into seven domains: nurse and non-medical prescribing outcomes; RN-led primary-care patient and system outcomes; interprofessional primary-care collaboration; chronic disease medication titration; patient experience and concordance; FP/NP coordination and service capacity; and implementation, safety, and regulatory conditions. To reduce interpretive bias, the synthesis distinguished findings directly reported by included studies, cross-domain interpretations, and proposed practice implications.

## 3. Results

### 3.1. Source Selection

**Figure 1:**
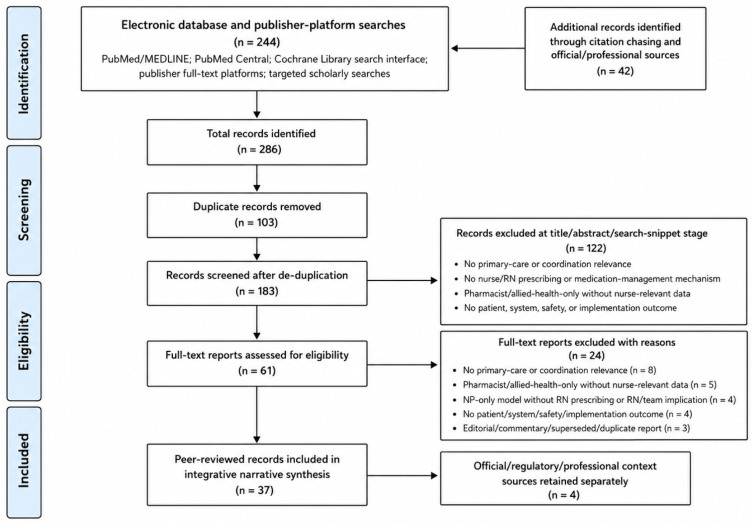
Search and selection flow diagram for identification, screening, eligibility assessment, and inclusion in the integrative review. Official/regulatory/professional sources were retained separately and were not counted as intervention-effect studies.

The search identified 286 records: 244 from database and publisher-platform searches and 42 from citation chasing and official/professional sources. After removal of 103 duplicates, 183 records were screened. One hundred and twenty-two records were excluded at title/abstract or search-snippet stage because they lacked primary-care relevance, did not address nurse or RN prescribing/medication-management mechanisms, were pharmacy-only or allied-health-only without nurse-relevant data, or did not report patient, system, safety, or implementation outcomes. Sixty-one reports were assessed for eligibility; 24 were excluded with reasons. Thirty-seven peer-reviewed records were included in the integrative narrative synthesis, and four official regulatory/professional sources were retained separately for context. The source-selection process is shown in Figure 1.

The numerical source-selection counts are summarized in Table 2.

### 3.2. Characteristics of Included Evidence

The included evidence comprised Cochrane and systematic reviews of nurse or non-medical prescribing, qualitative syntheses of implementation barriers and facilitators, RN-led primary-care systematic reviews, interprofessional collaboration reviews, disease-specific medication titration systematic reviews, patient-experience studies, and primary studies of nurse practitioner/family physician collaboration in primary care. Thirty records were review-level or evidence-synthesis sources, while seven were primary, mixed-methods, or patient-experience studies. Most high-level evidence therefore came from evidence syntheses rather than single primary studies. This was useful for mapping the field but increased indirectness and created the potential for overlap and amplification of biases from prior reviews. The main evidence domains, key sources, and interpretation for coordinated RN prescribing are summarized in Table 3.

### 3.3. Nurse and Non-Medical Prescribing Outcomes

The most pertinent prescribing evidence was from non-medical prescribing reviews. In a review of 46 studies, Weeks et al. reported that non-medical prescribers, including nurses and pharmacists, had outcomes similar to usual medical prescribers on a number of measures [13]. Reported outcomes included systolic blood pressure, LDL cholesterol, and HbA1c; medication adherence, patient satisfaction, and health-related quality of life were also similar or favourable [13]. However, not all studies reported on adverse events and resource use. This limits conclusions regarding medication safety and economic impact.

Gielen et al. proposed that nurse prescribing is largely comparable to that of physicians and could positively affect a number of clinical and satisfaction outcomes [14]. Bhanbhro et al. and Nuttall examined nurse prescribing in primary care. Access, timeliness, efficiency, professional confidence, and acceptance were common themes, though evidence at the patient level and for specific model implementation was limited [1,2].

### 3.4. RN-Led Primary Care and Access Mechanisms

Systematic reviews of RN-led primary care show that RNs already contribute meaningfully to patient and system outcomes. Lukewich et al. identified improvements across patient-outcome categories including blood pressure, glycemic control, self-efficacy, health behaviours, tobacco use, and patient satisfaction [16]. A companion review found that RN-led care affects medication management, triage, chronic disease management, sexual health, preventive care, education, and self-management support [17]. Norful et al. also found that integrating RNs into primary-care teams can increase access and improve care coordination [18].

The findings do not prove that RN prescribing itself is the causal mechanism in each case. They show, however, that RNs are already functioning in primary-care activities adjacent to prescribing, including assessment, follow-up, education, chronic disease monitoring, triage, and medication-related support. RN prescribing may therefore represent an extension of existing RN activity where regulatory authority, training, clinical support tools, and escalation pathways are in place.

### 3.5. Interprofessional Collaboration and Team-Based Care

Reviews of nurse–physician substitution and interprofessional primary care have shown that outcomes are likely to depend on how roles are organized. Laurant et al. and Martinez-Gonzalez et al. found that nurse-led or nurse-substitution models can result in similar or better outcomes than physician care for several clinical and patient-experience endpoints, but with different resource use in the form of longer consultations, more return visits, or different supervision needs [19,20,21,22].

Interprofessional collaboration reviews provide another important coordination mechanism. Matthys et al. reported evidence that collaboration between primary-care physicians and nurses may improve outcomes such as blood pressure and patient satisfaction and hospitalization, and Bouton et al. reported positive effects of interprofessional collaboration particularly in cardiovascular-risk management [24,25]. The evidence therefore supports collaboration as a relevant pathway through which RN prescribing may operate, while still leaving uncertainty about the structure and intensity of collaboration required.

### 3.6. Chronic Disease Medication Titration

Hypertension and diabetes had the most robust prescribing-adjacent clinical evidence. The hypertension evidence suggests that nurse-led interventions are more likely to be successful when prescriptive authority or structured treatment algorithms are present [26,27]. Other systematic reviews support the short- and long-term potential of nurse-led interventions to manage blood pressure in primary-care settings [28,29,31]. Vay-Demouy et al. reviewed nurse-led interventions with prescriptive authority and reported significant decreases in systolic and diastolic blood pressure compared with usual physician-led care, but noted a small randomized-trial base and high heterogeneity [30].

For diabetes, evidence suggests that nurse-led titration or nurse-led clinics can achieve similar or improved glycemic outcomes compared with usual physician-led approaches [32,33,34,35]. These outcomes are most relevant to RN prescribing when the patient has a known diagnosis, stable or predictable clinical status, an evidence-based medication pathway, laboratory monitoring, and clear escalation criteria. They are not generalizable to all undifferentiated presentations or all high-risk prescribing decisions.

### 3.7. Patient Experience, Acceptability, and Concordance

Patient-experience evidence generally supports nurse prescribing when patients perceive the nurse as competent, communicative, and able to meet the clinical need. Weiss et al. found high satisfaction in consultations involving nurse prescribers, pharmacist prescribers, and general practitioners [36]. Courtenay et al. and Stenner et al. reported patient acceptability in dermatology and diabetes-related nurse prescribing contexts [37,38]. Latter et al. and Hobson et al. found that patient-centred communication and concordance were important in nurse and pharmacist prescribing consultations [39,40]. Shum et al. showed that nurse-led management of minor illness in general practice was acceptable and safe in a randomized-trial context [41].

Patient acceptability therefore appears conditional. It is strengthened when patients understand the nurse prescriber role, receive clear explanations, can access timely care, and retain a pathway to a family physician or NP when the problem is more complex than initially expected.

### 3.8. Family Physicians and Nurse Practitioners as Coordination Partners

The rationale for discussing family physicians and NPs together in this review is operational, not professional equivalence. Both may act as coordination, continuity, and escalation partners for RN prescribing pathways, particularly when diagnostic uncertainty, unstable multimorbidity, treatment failure, abnormal investigations, high-risk prescribing, or longitudinal medical decision-making is present. However, family physicians and NPs differ in training pathway, regulatory authority, scope of practice, diagnostic and prescribing autonomy, accountability structures, and jurisdiction-specific practice rules. These differences should be considered when adapting the proposed model to local policy and clinical governance.

Roots and MacDonald reported that NPs embedded in collaborative rural primary-care practices improved access and were associated with decreased emergency use and hospital admissions in the studied setting [42]. McMenamin et al. found that NP primary-care models for patients with multiple chronic conditions were generally associated with equivalent or better quality, similar or lower emergency department and hospitalization outcomes, and reduced or similar costs compared with models without NP involvement [43]. Jokelin et al. described the expanding evidence base for multidisciplinary primary-care teams, while emphasizing heterogeneity and the need to understand which professionals add which benefits [44].

### 3.9. Implementation, Safety, and Regulatory Conditions

Implementation evidence repeatedly shows that prescribing authority must be supported by education, mentoring, protocols, role clarity, workflow design, and leadership. Edwards et al. organized implementation issues into preparation, training, transition, and sustainment [3]. Xu et al. found that nurses with prescriptive authority experience barriers related to undervaluing of nurse prescribers and the need for supportive systems [4]. Zhang et al. identified legal constraints, organizational structure, prescribing education, competence, team cooperation, and leadership support as major barriers or facilitators [5]. Alberta regulatory and standards documents are consistent with this implementation evidence by emphasizing clinical support tools, competence, documentation, follow-up, collaboration, and referral [6,7,8].

The evidence therefore supports a safety-oriented interpretation: RN prescribing should not be treated as inherently safe or unsafe in isolation. Its safety depends on the clinical domain, patient selection, prescriber competence, shared records, clinical support tools, follow-up responsibility, monitoring of abnormal results, and timely access to FP/NP consultation when the patient falls outside the RN prescribing pathway.

### 3.10. Critical Appraisal and Confidence in Interpretation

The most reliable evidence for prescribing outcomes came from systematic reviews and meta-analyses of non-medical prescribing, nurse-led hypertension interventions, and nurse-led diabetes models. However, direct relevance to the precise coordinated RN prescriber–FP/NP model was limited because many studies combined nurses with pharmacists, included international prescriber roles that differ from Canadian RN prescribing, evaluated nurse-led care without prescribing authority, or focused on chronic disease algorithms rather than broad primary-care prescribing. Qualitative and implementation syntheses were highly relevant for understanding barriers, safeguards, and organizational conditions, but they cannot establish comparative effectiveness. Evidence for adverse events, diagnostic safety, long-term continuity, workload redistribution, and total cost was less complete than evidence for access, patient satisfaction, and intermediate clinical indicators. The evidence hierarchy used to interpret the included sources is summarized in Table 4, and the narrative confidence assessment is presented in Table 5.

The following narrative confidence summary was used to interpret the main findings without applying formal GRADE ratings.

## 4. Discussion

### 4.1. Principal Interpretation

The evidence reviewed here supports a cautious and clinically practical interpretation. Nurse and non-medical prescribing can achieve comparable or favourable outcomes in selected contexts, RN-led primary care can improve several patient and system outcomes, structured nurse-led medication titration can improve intermediate chronic disease indicators, and interprofessional collaboration can contribute to primary-care outcomes. However, these findings come from related evidence domains rather than from direct testing of a complete coordinated RN prescriber–FP/NP model. The exact model of an RN prescriber embedded in a coordinated team with family physicians and NPs has not been directly evaluated often enough to claim causal superiority over other models.

The most defensible conclusion is therefore not that coordinated RN prescribing has been proven superior, but that the model is a reasonable interpretation to test in future implementation research. It should be viewed as an evidence-informed service-design hypothesis: plausible and relevant to primary-care workforce planning, but not yet established by direct comparative trials.

### 4.2. Why Coordination Matters

Observed evidence shows that nurse prescribing and nurse-led care can improve or match selected outcomes, especially when interventions are structured and patients are appropriate for the pathway. Implementation evidence shows that training, legal clarity, organizational support, and role clarity influence success. Collaboration evidence suggests that team-based primary care may improve outcomes in areas such as blood pressure control, satisfaction, hospitalization, and cardiovascular-risk management.

The inferred connection is that coordination may guard against the foreseeable hazards of fragmented prescribing. Disconnected RN prescribing may improve convenience at the cost of duplicate therapy, incomplete medication reconciliation, missed diagnostic uncertainty, unclear laboratory follow-up, or loss of continuity. A coordinated model could reduce those risks through shared electronic medical records, common clinical support tools, same-day escalation rules, FP/NP chart-routing, team huddles, medication reconciliation, and audit. These safeguards have not been evaluated as a single intervention package in the literature reviewed, but they are consistent with the implementation and safety conditions identified across sources.

### 4.3. Practice and Policy Implications

For future implementation and evaluation, RN prescribing can be conceptualized as a team-based service pathway rather than an isolated professional function. Potential pathways are likely to be most defensible where care is well-defined, lower-risk, or protocolized, including selected contraception and sexual health care, immunization-related prescribing where legally permitted, smoking cessation, stable chronic disease medication titration, medication renewal, hypertension follow-up, diabetes monitoring, urinary or dermatologic minor conditions where locally protocolized, and diagnostic testing clearly tied to a care plan.

Family physicians and NPs can reasonably be conceptualized as coordination partners for RN-prescriber escalation and continuity support, but this does not make the roles interchangeable. Family physicians and NPs differ in education, clinical training, regulatory authority, prescribing scope, practice autonomy, and accountability arrangements, and these differences vary by jurisdiction. Local implementation should therefore define which clinician is responsible for escalation, diagnostic review, abnormal results, high-risk prescribing, and longitudinal follow-up. An illustrative synthesis-informed conceptual framework for coordinated RN prescribing is presented in Table 6.

### 4.4. Evidence Gaps

The central evidence gap is direct comparative evaluation of coordinated RN prescriber–FP/NP models. Most available evidence evaluates nurse prescribing broadly, non-medical prescribing by mixed professional groups, nurse-led chronic disease management, nurse substitution, or interprofessional collaboration. These bodies of evidence are relevant but indirect. Future studies should compare coordinated RN prescribing against usual physician/NP-led care, RN-led care without prescribing, and less integrated prescribing models, while measuring access, clinical outcomes, adverse events, diagnostic safety, continuity, patient experience, workload, and cost.

Safety and economic outcomes require particular attention. Evidence for blood pressure, HbA1c, LDL cholesterol, adherence, and satisfaction is more developed than evidence for adverse drug events, missed diagnoses, antibiotic stewardship, abnormal-result follow-up, continuity, total visit volume, clinician workload, and total cost. Without these outcomes, it remains difficult to determine whether access gains represent true system value or a redistribution of work and risk.

### 4.5. Strengths and Limitations

The strengths of this review include having an explicit question, a structured eligibility framework, a documented search approach, transparent source-selection counts, a structured data-charting process, AMSTAR 2- and CASP-informed critical appraisal, and a synthesis that separates direct findings from interpretation. It synthesized across multiple relevant bodies of evidence rather than taking a stand-alone, decontextualized view of RN prescribing.

The main limitation is indirectness. Few direct studies compare coordinated RN prescriber–FP/NP teams with uncoordinated RN prescribing or usual physician/NP care. A large proportion of the strongest evidence concerns non-medical prescribing generally, nurse-led chronic disease management, nurse–physician substitution, or interprofessional collaboration rather than the specific RN prescribing model now being implemented in some Canadian jurisdictions. Several reviews include NPs, pharmacists or international nurse prescribers whose scope of practice, reimbursement or role may differ from Alberta RN prescribing.

The search was structured but not exhaustive. Embase, CINAHL, Scopus and Web of Science were not independently searched as stand-alone sources. This may have missed nursing-specific qualitative studies, role-development papers, implementation reports, education-focused studies, health-services literature, conference-indexed studies, and interdisciplinary primary-care workforce literature. The likely effect is that the review may underrepresent nursing practice, leadership, education, and implementation perspectives more than large clinical-effectiveness trials, many of which are already captured through PubMed/MEDLINE, Cochrane, publisher searches, and the included systematic reviews.

Additional methodological limitations should also be acknowledged. The working review plan was internal and not publicly posted or registered in PROSPERO, Open Science Framework, or another repository, which precludes external verification of a priori decisions. Cohen’s kappa was not calculated because selection was resolved through discussion within an integrative review process rather than through fully blinded duplicate systematic-review screening. English-language restrictions and reliance on accessible records may have introduced language or retrieval bias. The evidence base spans heterogeneous health systems, regulatory models, reimbursement arrangements and professional scopes of practice, limiting direct transferability. Publication bias is possible because successful prescribing and team-based programmes may be more likely to be evaluated and published than unsuccessful initiatives.

Because many included sources were reviews of reviews or systematic reviews, this review may also amplify limitations already present in the underlying reviews, including overlap of primary studies, inconsistent outcome definitions, and selective publication of favourable programmes. Evidence on adverse events, diagnostic safety, continuity of care, workload redistribution and costs remains limited. Finally, because the synthesis is interpretive, the possibility of interpretive bias cannot be eliminated, although it was reduced by separating observed findings, cross-domain interpretation and practice implications.

### 4.6. Research Implications

Future studies should evaluate structured RN prescribing in primary care using prospective designs. Appropriate designs include stepped-wedge rollouts, interrupted time series, matched-comparator clinic comparisons, pragmatic hybrid effectiveness-implementation trials, and linked administrative data studies. Reports should clearly describe the prescribing model that was implemented, including RN education and authorization method, clinical support tools, eligible conditions, formulary restrictions, diagnostic-test authority, supervision or collaboration requirements, electronic medical record workflow, escalation rules, frequency of FP/NP review, pharmacist role, audit process, and safety-netting with patients.

Important outcomes are broader than visit volume. Future studies should evaluate same-day and next-day access, attachment and continuity, time to appropriate medication adjustment, blood pressure, HbA1c, LDL cholesterol, medication adherence, adverse drug events, diagnostic delay, abnormal-result follow-up, antibiotic stewardship, emergency department use, hospitalization, patient-reported experience, clinician workload, cost, and equity by age, sex, socioeconomic status, rurality, language, disability, and digital access.

## 5. Conclusions

RN prescribing may improve primary-care access and selected intermediate clinical outcomes when used in appropriate, defined, and supported pathways. The current evidence is consistent with further evaluation of a coordinated model in which RN prescribers work within primary-care teams that include family physicians and NPs, but it does not directly prove that this configuration is superior to alternatives. In such a model, the RN prescriber may manage defined medication and assessment pathways, while family physicians and NPs provide diagnostic support, complex decision-making, high-risk prescribing, continuity, and escalation.

For the proposed model to be safe and useful, it would need to be supported by shared records, clinical support tools, role clarity, patient-centred communication, follow-up responsibility, and routine safety evaluation. Direct comparative studies of coordinated RN prescriber–FP/NP models remain needed. Until such evidence is available, coordinated RN prescribing should be presented as an evidence-informed and testable primary-care workforce model rather than as a proven superior model.

## Figures and Tables

**Table 1:** Eligibility framework for the integrative review.

Domain	Included	Excluded
Population	Adults, families, primary-care patients, and clinicians in family practice, general practice, community primary care, nurse-led primary care, or comparable ambulatory settings.	Hospital-only, inpatient-only, emergency-only, or specialty-only sources without transferable primary-care pathway.
Intervention/exposure	RN prescribing; nurse independent or supplementary prescribing; non-medical prescribing with nurse data; protocol-guided medication titration; RN-led primary care; nurse–physician/NP collaboration; task shifting or substitution involving nurses.	Interventions without prescribing, medication management, primary-care team coordination, or nurse involvement.
Outcomes	Access, timeliness, continuity, BP, HbA1c, LDL cholesterol, adherence, satisfaction, concordance, safety, referrals, ED or hospital use, costs, workload, role clarity, training, and implementation barriers/facilitators.	Sources reporting only professional opinion without patient, system, safety, or implementation outcome.
Designs	Systematic, scoping, and rapid reviews; randomized or quasi-experimental studies; cohort and cross-sectional analyses; qualitative and mixed-methods studies; implementation studies; regulatory/professional context sources.	Editorials, abstracts without sufficient data, superseded policy documents, and non-English records where reliable interpretation was not possible.
Publication window	Core peer-reviewed evidence from 2000 to 30 March 2026; earlier or official sources retained only where necessary for method, regulation, or foundational context.	Sources outside the window unless required for regulatory or methodological context.

**Table 2:** Search and selection summary.

Selection Stage	n
Electronic database and publisher-platform records identified	244
Records identified through citation chasing and official/professional sources	42
Total records identified	286
Duplicate records removed	103
Records screened after de-duplication	183
Records excluded at title/abstract/search-snippet stage	122
Full-text reports assessed for eligibility	61
Full-text reports excluded with reasons	24
Peer-reviewed records included in integrative narrative synthesis	37
Official/regulatory/professional context sources retained separately	4

**Table 3:** Evidence domains and interpretation for coordinated RN prescribing.

Evidence Domain	Key Sources	Interpretation for This Review
Non-medical and nurse prescribing	Weeks et al. [13]; Gielen et al. [14]; Bhanbhro et al. [1]; Nuttall [2]; Noblet et al. [15]	Comparable or favourable outcomes are reported in selected contexts, but mixed prescriber groups and incomplete safety/cost reporting reduce directness.
Implementation of nurse prescribing	Edwards et al. [3]; Xu et al. [4]; Zhang et al. [5]	Training, legal clarity, role recognition, leadership, workflow support, and team cooperation influence implementation.
RN-led primary-care roles	Lukewich et al. [16,17]; Norful et al. [18]	RNs contribute to medication management, triage, chronic disease care, prevention, education, and selected outcomes.
Nurse substitution and task shifting	Laurant et al. [19]; Martinez-Gonzalez et al. [20,21,22]; Paier-Abuzahra et al. [23]	Nurse-led care can match or improve selected outcomes; resource use depends on role design, consultation length, follow-up, and supervision.
Interprofessional collaboration	Matthys et al. [24]; Bouton et al. [25]	Collaboration is a relevant mechanism, not proof that one RN-prescribing configuration is superior.
Hypertension and diabetes pathways	Clark et al. [26,27]; Stephen et al. [28]; Bulto et al. [29]; Vay-Demouy et al. [30]; Ito et al. [31]; Sharma et al. [32]; Wang et al. [33]; Crowe et al. [34]; Tabesh et al. [35]	The clearest clinical signal concerns structured titration with algorithms, follow-up, and escalation.
Patient experience and acceptability	Weiss et al. [36]; Courtenay et al. [37]; Stenner et al. [38]; Latter et al. [39]; Hobson et al. [40]; Shum et al. [41]	Patients generally accept nurse prescribing and nurse-led care when competence, communication, choice, and timely access are clear.
FP/NP coordination and multidisciplinary care	Roots and MacDonald [42]; McMenamin et al. [43]; Jokelin et al. [44]	FPs and NPs are relevant coordination partners for complexity, continuity, and escalation, although direct RN prescriber–FP/NP evidence remains sparse.

**Table 4:** Evidence hierarchy and narrative confidence in key interpretations.

Evidence Type	Contribution to Synthesis	Main Limitations
Meta-analyses and systematic reviews of non-medical/nurse prescribing, chronic disease titration, and nurse-led care	Highest contribution to clinical outcome interpretation	Several combine nurses with pharmacists, include international roles, or do not isolate RN prescribing.
Qualitative syntheses and implementation reviews	Highest contribution to role clarity, training, legal, organizational, leadership, and workflow safeguards	Useful for implementation and safety interpretation; not comparative effectiveness evidence.
Primary, mixed-methods, RCT, observational, and patient-experience studies	Support acceptability, consultation processes, minor illness care, and selected FP/NP collaboration contexts	Smaller number of studies, often condition-specific or setting-specific.
Regulatory and professional documents	Contextualize Alberta/Canadian RN prescribing authority and professional expectations	Not treated as intervention-effect evidence.

**Table 5:** Narrative confidence summary used to interpret the main findings.

Interpretation	Evidence Base	Main Limitations	Confidence
Nurse/non-medical prescribing can achieve comparable outcomes for BP, HbA1c, LDL cholesterol, adherence, satisfaction, and quality of life in selected contexts.	Review-level evidence; several meta-analyses/systematic reviews	Mixed prescriber groups; incomplete adverse-event and resource-use reporting	Moderate confidence in direction; limited directness to RN-only primary-care teams.
RN-led primary-care interventions improve selected patient and system outcomes.	Systematic reviews of RN roles in primary care	Interventions vary; prescribing authority not always isolated	Moderate confidence for RN contribution; cautious inference for prescribing-specific effect.
Interprofessional collaboration is relevant to the value and safety of RN prescribing.	Collaboration reviews and implementation evidence	Few direct trials of coordinated RN prescriber–FP/NP models	Moderate confidence as implementation mechanism, not proof of superiority.
Protocolized nurse-led medication titration improves hypertension and diabetes indicators.	Multiple systematic reviews and meta-analyses	Heterogeneity and pathway-specific applicability	Moderate confidence for stable chronic disease pathways with algorithms and follow-up.
Patients accept nurse prescribing and nurse-led care when competence, communication, and access are clear.	Patient-experience and mixed-methods evidence	Many studies observational, qualitative, or condition-specific	Moderate confidence for acceptability under supportive conditions.

**Table 6:** Illustrative synthesis-informed conceptual framework for coordinated RN prescribing in primary care.

Component	Function	Required Safeguards
Patient entry and triage	Identify low-risk prescribing pathway, chronic disease follow-up, renewal, sexual health, preventive care, or minor illness need.	Eligibility criteria, red flags, same-day FP/NP escalation, and shared EMR documentation.
RN prescriber role	Assess within clinical support tool; prescribe or renew eligible medicines; order eligible tests; educate; document goals and follow-up.	Prescribe only within authorization, competence, clinical support tool, and patient-specific suitability.
Family physician or NP role	Manage diagnosis and complexity; review exceptions; support chronic care plans; handle high-risk prescribing, unstable results, or treatment failure.	Define local responsibilities for escalation, review, continuity, and abnormal-result follow-up.
Escalation pathway	Provide consultation for red flags, diagnostic uncertainty, multimorbidity, pregnancy/frailty concerns, interaction risk, poor response, or abnormal tests.	Named FP/NP covering clinician, response-time targets, and after-hours plan.
Quality and safety monitoring	Audit selected RN prescriptions; track outcomes, adverse events, ED use, antibiotic stewardship, workload, and satisfaction.	Feedback loop to revise clinical support tools, education, and workflow.

Note: Table 6 is an illustrative conceptual framework derived from interpretive synthesis. It is not a clinical guideline, not a prescriptive implementation standard, and has not been empirically evaluated as a complete intervention package.

## Data Availability

No new data were created or analyzed in this study. Data sharing is not applicable to this article.

## Supplementary Materials

The following supporting information can be downloaded at https://www.mdpi.com/article/10.3390/healthcare14131899/s1, Supplementary File S1: Review approach, search strategy and limits; Supplementary File S2: Evidence-hierarchy summary; Supplementary File S3: Study-level methodological quality appraisal and evidence-contribution table; Supplementary File S4: Grouped full-text exclusions and selection-decision examples.

## Abbreviations

AMSTAR 2: A Measurement Tool to Assess Systematic Reviews 2; BP: Blood Pressure; CASP: Critical Appraisal Skills Programme; CNA: Canadian Nurses Association; CRNA: College of Registered Nurses of Alberta; ED: Emergency Department; EMR: Electronic Medical Record; FP: Family Physician; GP: General Practitioner; HbA1c: Glycated Hemoglobin; LDL: Low-Density Lipoprotein; NP: Nurse Practitioner; RN: Registered Nurse; SBP: Systolic Blood Pressure.

## References

[1] Bhanbhro S., Drennan V.M., Grant R., Harris R. (2011). Assessing the contribution of prescribing in primary care by nurses and professionals allied to medicine: A systematic review of literature. BMC Health Serv. Res..

[2] Nuttall D. (2018). Nurse prescribing in primary care: A metasynthesis of the literature. Prim. Health Care Res. Dev..

[3] Edwards J., Coward M., Carey N. (2022). Barriers and facilitators to implementation of non-medical independent prescribing in primary care in the UK: A qualitative systematic review. BMJ Open.

[4] Xu J., Qi L., Mao A. (2025). The barriers to nurses with prescriptive authority in exercising their prescriptive role: A systematic review and thematic synthesis of qualitative studies. Glob. Qual. Nurs Res..

[5] Zhang Q., Cao G., Duan X., Zhu R., Han S. (2025). Barriers and facilitators to implementation of nurse prescribing: A qualitative synthesis based on the Consolidated Framework for Implementation Research. J. Clin. Nurs..

[6] College of Registered Nurses of Alberta (2023). Registered Nurse Prescribing Schedule 1 Drugs and Ordering Diagnostic Tests: Guidelines.

[7] College of Registered Nurses of Alberta (2023). Registered Nurse Prescribing Schedule 1 Drugs and Ordering Diagnostic Tests: Requirements and Standards.

[8] College of Registered Nurses of Alberta (2023). Competencies for Registered Nurse Prescribing Schedule 1 Drugs and Ordering Diagnostic Tests.

[9] Canadian Nurses Association (2015). Framework for Registered Nurse Prescribing in Canada.

[10] Whittemore R., Knafl K. (2005). The integrative review: Updated methodology. J. Adv. Nurs..

[11] Shea B.J., Reeves B.C., Wells G., Thuku M., Hamel C., Moran J., Moher D., Tugwell P., Welch V., Kristjansson E. (2017). AMSTAR 2: A critical appraisal tool for systematic reviews that include randomised or non-randomised studies of healthcare interventions. BMJ.

[12] Critical Appraisal Skills Programme (2024). CASP Checklists.

[13] Weeks G., George J., Maclure K., Stewart D. (2016). Non-medical prescribing versus medical prescribing for acute and chronic disease management in primary and secondary care. Cochrane Database Syst. Rev..

[14] Gielen S.C., Dekker J., Francke A.L., Mistiaen P., Kroezen M. (2014). The effects of nurse prescribing: A systematic review. Int. J. Nurs. Stud..

[15] Noblet T., Marriott J., Graham-Clarke E., Shirley D., Rushton A. (2018). Clinical and cost-effectiveness of non-medical prescribing: A systematic review of randomised controlled trials. PLoS ONE.

[16] Lukewich J., Martin-Misener R., Norful A.A., Poitras M.-E., Bryant-Lukosius D., Asghari S., Marshall E.G., Mathews M., Swab M., Ryan D. (2022). Effectiveness of registered nurses on patient outcomes in primary care: A systematic review. BMC Health Serv. Res..

[17] Lukewich J., Asghari S., Marshall E.G., Mathews M., Swab M., Tranmer J., Bryant-Lukosius D., Martin-Misener R., Norful A.A., Ryan D. (2022). Effectiveness of registered nurses on system outcomes in primary care: A systematic review. BMC Health Serv. Res..

[18] Norful A.A., de Jacq K., Carlino R., Poghosyan L. (2017). Utilization of registered nurses in primary care teams: A systematic review. Int. J. Nurs. Stud..

[19] Laurant M., van der Biezen M., Wijers N., Watananirun K., Kontopantelis E., van Vught A.J.A.H. (2018). Nurses as substitutes for doctors in primary care. Cochrane Database Syst. Rev..

[20] Martinez-Gonzalez N.A., Djalali S., Tandjung R., Huber-Geismann F., Markun S., Wensing M., Rosemann T. (2014). Substitution of physicians by nurses in primary care: A systematic review and meta-analysis. BMC Health Serv. Res..

[21] Martinez-Gonzalez N.A., Tandjung R., Djalali S., Huber-Geismann F., Markun S., Wensing M., Rosemann T. (2014). Effects of physician-nurse substitution on clinical parameters: A systematic review and meta-analysis. PLoS ONE.

[22] Martinez-Gonzalez N.A., Djalali S., Tandjung R., Huber-Geismann F., Markun S., Wensing M., Rosemann T. (2015). Impact of physician-nurse substitution on health service resource use and costs: A systematic review. Med. Care Res. Rev..

[23] Paier-Abuzahra M., Posch N., Jeitler K., Semlitsch T., Radl-Karimi C., Spary-Kainz U., Horvath K., Siebenhofer A. (2024). Effects of task-shifting from primary care physicians to nurses: An overview of systematic reviews. Hum. Resour. Health.

[24] Matthys E., Remmen R., Van Bogaert P. (2017). An overview of systematic reviews on the collaboration between physicians and nurses and the impact on patient outcomes: What can we learn in primary care?. BMC Fam. Pract..

[25] Bouton C., Journeaux M., Jourdain M., Angibaud M., Huon J.-F., Rat C. (2023). Interprofessional collaboration in primary care: What effect on patient outcomes? A systematic literature review. BMC Prim. Care..

[26] Clark C.E., Smith L.F.P., Taylor R.S., Campbell J.L. (2010). Nurse led interventions to improve control of blood pressure in people with hypertension: Systematic review and meta-analysis. BMJ.

[27] Clark C.E., Smith L.F.P., Taylor R.S., Campbell J.L. (2011). Nurse-led interventions used to improve control of high blood pressure in people with diabetes: A systematic review and meta-analysis. Diabet. Med..

[28] Stephen C., Halcomb E., Fernandez R., McInnes S., Batterham M., Zwar N. (2022). Nurse-led interventions to manage hypertension in general practice: A systematic review and meta-analysis. J. Adv. Nurs..

[29] Bulto L.N., Roseleur J., Noonan S., Pinero de Plaza M.A., Champion S., Dafny H.A., Pearson V., Nesbitt K., Gebremichael L.G., Beleigoli A. (2024). Effectiveness of nurse-led interventions versus usual care to manage hypertension and lifestyle behaviour: A systematic review and meta-analysis. Eur. J. Cardiovasc. Nurs..

[30] Vay-Demouy J., Lelong H., Blacher J. (2025). Impact of nurse-led interventions with prescriptive authority on blood pressure control in hypertension management: A systematic review and meta-analysis. BMC Nurs..

[31] Ito M., Tajika A., Toyomoto R., Imai H., Sakata M., Honda Y., Kishimoto S., Fukuda M., Horinouchi N., Sahker E. (2024). The short- and long-term efficacy of nurse-led interventions for improving blood pressure control in people with hypertension in primary care settings: A systematic review and meta-analysis. BMC Prim. Care.

[32] Sharma S.K., Thakur K., Kant R., Mudgal S.K. (2021). Impact of nurse-led titration versus physician prescription of hypoglycaemic agents on HbA1c level in type 2 diabetes patients: A systematic review and meta-analysis of randomized controlled trials. Cureus.

[33] Wang Q., Shen Y., Chen Y., Li X. (2019). Impacts of nurse-led clinic and nurse-led prescription on hemoglobin A1c control in type 2 diabetes: A meta-analysis. Medicine.

[34] Crowe M., Jones V., Stone M.-A., Coe G. (2019). The clinical effectiveness of nursing models of diabetes care: A synthesis of the evidence. Int. J. Nurs. Stud..

[35] Tabesh M., Magliano D.J., Koye D.N., Shaw J.E. (2018). The effect of nurse prescribers on glycaemic control in people with type 2 diabetes: A systematic review and meta-analysis. Int. J. Nurs. Stud..

[36] Weiss M.C., Sutton J., Adams C. (2015). Exploring innovation in nurse and pharmacist prescribing: A quantitative evaluation of consultation types and patient experience. Prim. Health Care Res. Dev..

[37] Courtenay M., Carey N., Stenner K., Lawton S., Peters J. (2011). Patients views of nurse prescribing: Effects on care, concordance and medicine taking. Br. J. Dermatol..

[38] Stenner K., Courtenay M., Carey N. (2011). Consultations between nurse prescribers and patients with diabetes in primary care: A qualitative study of patient views. Int. J. Nurs. Stud..

[39] Latter S., Maben J., Myall M., Young A. (2007). Perceptions and practice of concordance in nurses prescribing consultations: Findings from a national questionnaire survey and case studies of practice in England. Int. J. Nurs. Stud..

[40] Hobson R.J., Scott J., Sutton J. (2010). Pharmacists and nurses as independent prescribers: Exploring the patients perspective. Fam. Pract..

[41] Shum C., Humphreys A., Wheeler D., Cochrane M.A., Skoda S., Clement S. (2000). Nurse management of patients with minor illnesses in general practice: Multicentre, randomised controlled trial. BMJ.

[42] Roots A., MacDonald M. (2014). Outcomes associated with nurse practitioners in collaborative practice with general practitioners in rural settings in Canada: A mixed methods study. Hum. Resour. Health.

[43] McMenamin A., Turi E., Schlak A., Poghosyan L. (2023). A systematic review of outcomes related to nurse practitioner-delivered primary care for multiple chronic conditions. Med. Care Res. Rev..

[44] Jokelin E., Karreinen S., Mustonen E., Torkki P. (2025). Clinical and economic outcomes of multidisciplinary team members in primary care: A scoping review. BMC Health Serv. Res..

