# Supplementary material for: Collaborative Primary-Care Workforce Models: An Integrative Review of Evidence Informing RN Prescriber Integration with Family Physicians and Nurse Practitioners
Source: Healthcare (Basel). 2026 Jun 30;14(13):1899. doi: 10.3390/healthcare14131899 (PMC13361783; doi:10.3390/healthcare14131899)
Supplement: Supplementary file 1 [file healthcare-14-01899-s001.zip › healthcare-4379077-supplementary.pdf]

Supplementary Files S1-S4

Review Approach, Search Strategy, Evidence Hierarchy, Methodological Quality/Risk-of-Bias Considerations, and Source-Selection Details

Collaborative Primary Care Workforce Models: An Integrative Review of Evidence Informing RN Prescriber Integration with Family Physicians and Nurse Practitioners

Tomasz Karczewski, Dawid Karczewski, Merjorie M. A. Pinero, and Avni K. Patel

S1. Review Approach, Search Strategy, and Limits

Table S1. Review approach, search strategy, search date, and limits.

Source/search type	Strategy, date, or limit
Review approach and working plan	Integrative review designed to synthesize heterogeneous evidence relevant to RN prescribing within team-based primary care. A working review plan specified the review question, eligibility framework, information sources, source-selection process, data-charting variables, appraisal approach, and synthesis domains. The plan guided the review process but was internal rather than publicly posted or registered in PROSPERO, Open Science Framework, or another repository.
Final search/consultation date	All reported electronic, targeted scholarly, citation, regulatory, and professional source searches were last completed or checked on 30 March 2026.
PubMed/MEDLINE core	("nurse prescribing"[tiab] OR "registered nurse prescribing"[tiab] OR "non-medical prescribing"[tiab] OR "nurse prescriber"[tiab] OR "nurse-led"[tiab] OR "medication titration"[tiab] OR "task shifting"[tiab]) AND ("primary care"[tiab] OR "family practice"[tiab] OR "general practice"[tiab] OR community[tiab] OR ambulatory[tiab]) AND (physician[tiab] OR "family physician"[tiab] OR "general practitioner"[tiab] OR "nurse practitioner"[tiab] OR interprofessional[tiab] OR collaboration[tiab] OR team[tiab]) AND (access[tiab] OR safety[tiab] OR satisfaction[tiab] OR adherence[tiab] OR "blood pressure"[tiab] OR HbA1c[tiab] OR prescribing[tiab] OR implementation[tiab]) AND ("2000/01/01"[Date - Publication] : "2026/03/30"[Date - Publication]).
Short recall searches	"registered nurse prescribing primary care"; "nurse prescribing family practice"; "non-medical prescribing versus medical prescribing systematic review"; "nurse-led medication titration hypertension primary care"; "nurse-led diabetes titration physician systematic review"; "nurse physician collaboration primary care systematic review";

	"registered nurses primary care outcomes systematic review"; "nurse prescribing barriers facilitators qualitative synthesis".
Official/professional sources	College of Registered Nurses of Alberta RN prescribing standards, guidelines, and competencies; Canadian Nurses Association RN prescribing framework.
Limits	Core peer-reviewed window 2000 to 30 March 2026; official and methodological sources retained when directly necessary; English-language records or records with sufficient English-language information for reliable interpretation.
Important limitation	The search was structured but not exhaustive. Embase, CINAHL, Scopus, and Web of Science were not independently searched as stand-alone databases. A complementary CINAHL search was considered but could not be completed in an independently auditable manner for this revision. This limitation may particularly affect retrieval of nursing-specific qualitative literature, role-development studies, education-focused records, implementation papers, and professional-practice research; the likely implications are addressed in the main manuscript.

S2. Evidence Hierarchy and Contribution to Interpretation

Table S2. Evidence hierarchy used to weight evidence in the integrative synthesis. This hierarchy is descriptive and was used to guide interpretation; it is not a formal GRADE assessment.

Evidence level	Evidence type	Use in synthesis	Limitations
A	Meta-analyses and systematic reviews of nurse/non-medical prescribing, chronic disease titration, nurse-led care, or task shifting	Highest contribution to clinical-outcome interpretation	Several combine nurses with pharmacists, include international roles, or do not isolate RN prescribing within FP/NP teams.
B	Qualitative syntheses, implementation syntheses, scoping reviews, and overviews of reviews	High contribution to understanding barriers, safeguards, role clarity, implementation, and context	Cannot establish comparative effectiveness; may amplify limitations of included primary studies.
C	Primary quantitative, qualitative, mixed-methods, patient-experience, and randomized studies	Useful for patient experience, acceptability, feasibility, and selected service outcomes	Often condition-specific, older, context-dependent, or not designed to test the proposed model.

D	Regulatory and professional sources	Contextual interpretation of scope, standards, authorization, and implementation requirements	Not treated as intervention-effect evidence unless reporting original data.

S3. Study-Level Methodological Quality and Risk-of-Bias Considerations

The appraisal used AMSTAR 2-informed considerations for review-level evidence and CASP-informed considerations for qualitative, mixed-methods, observational, and primary studies. Ratings are descriptive and indicate the confidence contribution of each source to this integrative synthesis; they are not formal GRADE ratings. The table should be read as a structured methodological-quality and risk-of-bias consideration table rather than as a quantitative risk-of-bias score.

Table S3. Study-level methodological quality/risk-of-bias considerations and contribution to synthesis.

**Source**	**Evidence type**	**Appraisal lens**	Main methodological quality/risk-of-bias considerations	**Directness to review question**	**Contribution**
Weeks et al. [8]	Cochrane review of non-medical prescribing	AMSTAR 2-informed	Transparent review methods and broad comparative evidence; mixed nurse/pharmacist prescriber groups and incomplete adverse-event/resource-use reporting.	Moderate: strong for non-medical prescribing generally, indirect for RN-FP/NP model.	Core evidence for prescribing outcomes.
Gielen et al. [9]	Systematic review of nurse prescribing	AMSTAR 2-informed	Relevant nurse-specific review; older evidence and heterogeneous settings.	Moderate: relevant to nurse prescribing, indirect for team configuration.	Supports general effectiveness and acceptability.
Bhanbhro et al. [10]	Systematic review of prescribing by nurses/allied professionals in primary care	AMSTAR 2-informed	Primary-care relevance; includes professionals allied to medicine and older evidence.	Moderate to low: useful but not RN-specific throughout.	Supports access and primary-care implementation context.
Nuttall [11]	Metasynthesis of nurse prescribing in primary care	CASP/qualitative synthesis-informed	Primary-care focus; qualitative interpretive synthesis with context dependence.	Moderate: relevant to implementation and acceptance.	Informs barriers, confidence, and role development.

Noblet et al. [12]	Systematic review of RCTs in mental health non-medical prescribing	AMSTAR 2-informed	Trial focus but narrow clinical domain and limited cost evidence.	Low to moderate: mental health-specific and indirect.	Highlights domain-specific safeguards.
Edwards et al. [13]	Qualitative systematic review of implementation in UK primary care	CASP-informed	Strong implementation relevance; UK context may limit transferability.	Moderate to high for implementation factors.	Supports preparation, training, transition, and sustainment domains.
Xu et al. [14]	Qualitative thematic synthesis of barriers for nurses with prescriptive authority	CASP-informed	Recent qualitative synthesis; not limited to Canadian primary care.	Moderate: direct to nurse prescriptive role but context-varied.	Informs undervaluing, role support, and organizational barriers.
Zhang et al. [15]	Qualitative synthesis using CFIR	CASP-informed	Recent framework-based synthesis; international heterogeneity.	Moderate: strong for implementation constructs, indirect for FP/NP coordination.	Supports legal, organizational, education, and leadership safeguards.
Lukewich et al. [16]	Systematic review of RN effects on patient outcomes in primary care	AMSTAR 2-informed	Strong RN primary-care relevance; prescribing effects not isolated.	Moderate: direct to RN primary care, indirect to prescribing.	Supports RN contribution to patient outcomes.
Lukewich et al. [17]	Systematic review of RN effects on system outcomes in primary care	AMSTAR 2-informed	Strong RN role relevance; system outcomes vary by intervention.	Moderate: direct to RN primary care, indirect to prescribing.	Supports medication management, triage, prevention, and care coordination.
Norful et al. [18]	Systematic review of registered nurses in primary-care teams	AMSTAR 2-informed	Relevant to team integration; heterogeneous primary studies.	Moderate: direct to RN team roles, indirect to prescribing.	Supports access and coordination mechanisms.
Laurant et al. [19]	Cochrane review of nurses as substitutes for doctors in primary care	AMSTAR 2-informed	High methodological credibility; substitution not equivalent to RN prescribing.	Moderate: strong for nurse-led primary care, indirect for RN prescribing.	Supports careful interpretation of nurse-led care and resource use.
Martinez-Gonzalez et al. [20]	Systematic review/meta-analysis of physician-nurse substitution	AMSTAR 2-informed	Quantitative synthesis but substantial heterogeneity and role variation.	Moderate: relevant to task shifting, indirect to RN prescribing.	Supports outcomes of nurse substitution.

Martinez-Gonzalez et al. [21]	Systematic review/meta-analysis of clinical parameters	AMSTAR 2-informed	Clinical endpoints relevant but not prescribing-specific in all studies.	Moderate: relevant to nurse-led chronic care.	Supports selected clinical equivalence/improvement.
Martinez-Gonzalez et al. [22]	Systematic review of resource use and costs	AMSTAR 2-informed	Economic estimates depend on consultation length, follow-up and wage structure.	Moderate to low: useful but context-sensitive.	Informs caution around cost and workload claims.
Paier-Abuzahra et al. [23]	Overview of systematic reviews of task shifting	AMSTAR 2-informed umbrella considerations	Broad task-shifting scope; direct applicability varies.	Moderate to low: broad workforce evidence.	Supports contextualization of nursing workforce redesign.
Matthys et al. [24]	Overview of systematic reviews on physician-nurse collaboration	AMSTAR 2-informed	Focuses collaboration broadly and outcomes vary.	Moderate: relevant to primary-care collaboration, indirect to RN prescribing.	Supports coordination as outcome mechanism.
Bouton et al. [25]	Systematic review of interprofessional collaboration in primary care	AMSTAR 2-informed	Heterogeneous interventions and outcomes.	Moderate: direct to collaboration, indirect to RN prescribing.	Supports team-based cardiovascular-risk and primary-care outcomes.
Clark et al. [26]	Systematic review/meta-analysis of nurse-led hypertension interventions	AMSTAR 2-informed	Older trials but clinically relevant and quantitative.	Moderate: direct to nurse-led BP care, indirect to RN prescribing unless authority specified.	Supports algorithmic hypertension pathways.
Clark et al. [27]	Systematic review/meta-analysis of nurse-led BP control in diabetes	AMSTAR 2-informed	Disease-specific and older evidence.	Moderate: relevant to chronic disease titration.	Supports structured chronic disease follow-up.
Stephen et al. [28]	Systematic review/meta-analysis of nurse-led hypertension in general practice	AMSTAR 2-informed	Primary-care relevant; intervention variation remains.	Moderate: good directness to general practice, partial to prescribing.	Supports nurse-led hypertension outcomes.
Bulto et al. [29]	Systematic review/meta-analysis of nurse-led hypertension/lifestyle care	AMSTAR 2-informed	Recent synthesis; mixed intervention components.	Moderate: relevant to hypertension/lifestyle management.	Supports effectiveness of nurse-led interventions.

Vay-Demouy et al. [30]	Systematic review/meta-analysis of nurse-led interventions with prescriptive authority	AMSTAR 2-informed	Highly relevant to prescribing authority; small RCT base and heterogeneity.	Moderate: direct to nurse prescriptive authority, condition-specific.	Strong support for cautious chronic disease prescribing pathways.
Ito et al. [31]	Systematic review/meta-analysis of nurse-led BP interventions	AMSTAR 2-informed	Recent evidence; primary-care focus, but prescribing not always isolated.	Moderate: direct to nurse-led BP care.	Supports short- and long-term BP management.
Sharma et al. [32]	Systematic review/meta-analysis of nurse-led titration vs physician prescription in type 2 diabetes	AMSTAR 2-informed	Disease-specific RCT evidence; scope and setting variation.	Moderate: relevant to glycemic medication titration.	Supports diabetes titration pathways.
Wang et al. [33]	Meta-analysis of nurse-led clinic and nurse-led prescription for HbA1c	AMSTAR 2-informed	Heterogeneous diabetes models and settings.	Moderate: diabetes-specific and partially prescribing-relevant.	Supports glycemic outcome evidence.
Crowe et al. [34]	Synthesis of nursing models of diabetes care	AMSTAR 2-informed	Broad nursing models; prescribing effect not always separable.	Moderate to low: diabetes care relevant but indirect.	Supports chronic disease care models.
Tabesh et al. [35]	Systematic review/meta-analysis of nurse prescribers and glycemic control	AMSTAR 2-informed	Relevant to nurse prescribers; clinical context limited to diabetes.	Moderate: direct to nurse prescribing in diabetes.	Supports diabetes prescribing/titration evidence.
Weiss et al. [36]	Quantitative evaluation of consultation types and patient experience	CASP/observational-informed	Observational patient-experience study; not designed for effectiveness.	Moderate to low: patient experience direct, outcome indirect.	Supports satisfaction and consultation acceptability.
Courtenay et al. [37]	Patient views of nurse prescribing in dermatology	CASP-informed	Condition-specific patient-experience evidence.	Moderate: direct to nurse prescribing, narrow scope.	Supports acceptability and concordance.
Stenner et al. [38]	Qualitative study of nurse prescriber consultations in diabetes	CASP-informed	Qualitative and disease-specific; useful for patient perspective.	Moderate: direct to nurse prescribing in diabetes.	Supports communication and patient trust themes.

Latter et al. [39]	National questionnaire survey and case studies of nurse prescribing consultations	CASP/mixed-methods-informed	Older data; mixed survey and case-study methods.	Low to moderate: relevant to consultation quality.	Informs concordance and patient-centered communication.
Hobson et al. [40]	Patient perspective on pharmacist and nurse independent prescribers	CASP-informed	Patient-experience focus; mixed professional groups.	Low to moderate: relevant to acceptability, indirect for RN team model.	Supports patient perspective on prescriber roles.
Shum et al. [41]	Multicentre randomized trial of nurse management of minor illness	Trial risk-of-bias principles	Randomized design but dated and not primarily RN prescribing.	Moderate: direct to nurse-led minor illness, indirect to prescribing.	Supports minor-illness pathway feasibility.
Roots and MacDonald [42]	Mixed-methods study of NP-GP collaborative rural practice in Canada	CASP/mixed-methods-informed	Canadian relevance; NP rather than RN prescriber model.	Moderate to low: coordination partner evidence, indirect to RN prescribing.	Supports FP/NP collaboration and access context.
McMenamin et al. [43]	Systematic review of NP-delivered primary care for multiple chronic conditions	AMSTAR 2-informed	NP model evidence; not RN prescribing.	Low to moderate: relevant to NP coordination role.	Supports NP role in primary-care capacity and chronic care.
Jokelin et al. [44]	Scoping review of multidisciplinary teams in primary care	Scoping review appraisal considerations	Recent and broad; team composition and outcomes vary.	Low to moderate: broad multidisciplinary evidence, indirect to RN prescribing.	Supports broader team-based workforce context.

S4. Full-Text Exclusion Categories

Table S4. Grouped full-text exclusions and selection-decision examples.

**Reason for exclusion**	**n**	**Selection-decision example**
No primary-care or coordination relevance	8	Hospital-only, emergency-only, or specialty-only intervention without a transferable primary-care prescribing or team pathway.
Pharmacist/allied-health-only non-medical prescribing without nurse-relevant data	5	Non-medical prescribing record where nurse results could not be separated or interpreted.

NP-only model without RN prescribing or RN/team implication	4	NP primary-care record retained only when it informed FP/NP coordination or escalation for RN prescribing; otherwise excluded.
No patient/system/safety/implementation outcome	4	Role description, education-only source, or scope discussion without outcome, safety, or implementation data.
Editorial/commentary/superseded/duplicate report	3	Opinion piece, abstract without sufficient data, superseded guidance, or duplicate record.
